# Tiling resolution array CGH and high density expression profiling of urothelial carcinomas delineate genomic amplicons and candidate target genes specific for advanced tumors

**DOI:** 10.1186/1755-8794-1-3

**Published:** 2008-01-31

**Authors:** Markus Heidenblad, David Lindgren, Tord Jonson, Fredrik Liedberg, Srinivas Veerla, Gunilla Chebil, Sigurdur Gudjonsson, Åke Borg, Wiking Månsson, Mattias Höglund

**Affiliations:** 1 Department of Clinical Genetics, Lund University Hospital, SE-221 85 Lund, Sweden; 2 Department of Urology, Lund University Hospital, SE-221 85 Lund, Sweden; 3 Department of Pathology and Cytology, Helsingborg Hospital AB, SE-251 87 Helsingborg, Sweden; 4 Department of Oncology, Lund University Hospital, SE-221 85 Lund, Sweden

## Abstract

**Background:**

Urothelial carcinoma (UC) is characterized by nonrandom chromosomal aberrations, varying from one or a few changes in early-stage and low-grade tumors, to highly rearranged karyotypes in muscle-invasive lesions. Recent array-CGH analyses have shed further light on the genomic changes underlying the neoplastic development of UC, and have facilitated the molecular delineation amplified and deleted regions to the level of specific candidate genes. In the present investigation we combine detailed genomic information with expression information to identify putative target genes for genomic amplifications.

**Methods:**

We analyzed 38 urothelial carcinomas by whole-genome tiling resolution array-CGH and high density expression profiling to identify putative target genes in common genomic amplifications. When necessary expression profiling was complemented with Q-PCR of individual genes.

**Results:**

Three genomic segments were frequently and exclusively amplified in high grade tumors; 1q23, 6p22 and 8q22, respectively. Detailed mapping of the 1q23 segment showed a heterogeneous amplification pattern and no obvious commonly amplified region. The 6p22 amplicon was defined by a 1.8 Mb core region present in all amplifications, flanked both distally and proximally by segments amplified to a lesser extent. By combining genomic profiles with expression profiles we could show that amplification of *E2F3, CDKAL1*, *SOX4*, and *MBOAT1 *as well as *NUP153, AOF1, FAM8A1 *and *DEK *in 6p22 was associated with increased gene expression. Amplification of the 8q22 segment was primarily associated with *YWHAZ (14-3-3-zeta) *and *POLR2K *over expression. The possible importance of the *YWHA *genes in the development of urothelial carcinomas was supported by another recurrent amplicon paralogous to 8q22, in 2p25, where increased copy numbers lead to enhanced expression of *YWHAQ *(*14-3-3-theta*). Homozygous deletions were identified at 10 different genomic locations, most frequently affecting *CDKN2A/CDKN2B *in 9p21 (32%). Notably, the latter occurred mutually exclusive with 6p22 amplifications.

**Conclusion:**

The presented data indicates 6p22 as a composite amplicon with more than one possible target gene. The data also suggests that amplification of 6p22 and homozygous deletions of 9p21 may have complementary roles. Furthermore, the analysis of paralogous regions that showed genomic amplification indicated altered expression of *YWHA *(14-3-3) genes as important events in the development of UC.

## Background

Urothelial carcinoma (UC) is characterized by nonrandom chromosomal aberrations, varying from one or a few changes in early-stage and low-grade tumors, to massively rearranged karyotypes in muscle-invasive lesions [1]. Rearrangements of chromosome 9, resulting in loss of chromosome 9 material, are the most common cytogenetic alterations and are seen in close to half of the cases. Loss of material from chromosome arm 11p, and gains of chromosome 7 and chromosome arm 1q seem to be early but secondary changes. UC is also characterized by a strong association between tumor stage/grade and karyotypic complexity, indicating that progressive accumulation of genetic alterations is the driving force behind multi-step bladder carcinogenesis [2]. Cytogenetic data have been corroborated by comparative genomic hybridization (CGH) investigations [3-5], which in addition have been valuable for identification of high-level amplifications. Recent array CGH analyses of bladder tumors [6,7] and cell lines [8], based on non-tiling bacterial artificial chromosome (BAC) arrays have shed further light on the genomic changes underlying neoplastic development. These studies have facilitated the molecular delineation of additional amplified and deleted regions to the level of specific candidate genes. Recurrent high-level local amplifications have so far been reported in many genomic regions, including 1q21-24, 3p22-24, 6p22, 8q21-22, 10p13-14, 11q13, 12q13-15, and 17q21 [3-8]. To provide a more complete and detailed map of previously observed amplified and deleted segments, and to identify new copy number aberrations (CNAs) of importance to urothelial tumorigenesis, we used whole-genome tiling-resolution array CGH based on a 32 k BAC clone set to analyze 38 primary urothelial carcinomas. In order to allow a comprehensive analysis also of transcriptional effects of the identified CNAs, the copy number analysis was complemented with expression profiling using high-density (36 k) oligonucleotide micro arrays. With this approach we present genomic borders and putative target genes for 9 recurrent amplifications, some of which specific for advanced tumors, and for 7 previously not described homozygous deletions, of which one is recurrent. We also studied the pattern of CNA occurrence, and show that 6p22 amplifications are not independent of homozygous deletions in 9p21, and that amplification of paralogous segments in 8q22 and 2p25 appear to act complementary and may have equivalent biological outcomes as they both result in increased expression of members of the *YWHA *genes, encoding 14-3-3 proteins.

## Methods

### Patients and tissue

Tumors were collected by cold-cup biopsies from the exophytic part of the bladder in patients undergoing resection at the University Hospital of Lund, Sweden, between 2001 and 2004 and kept at -80C until further use. Sample quality was evaluated by histology. Altogether 10 Ta, 9 T1, and 19 muscle-invasive (T2-T4) tumors were included in the study. Of these 15 were low grade (grades G1 and G2) and 23 high grade (G3) tumors. Tumor pathology, based on transurethral and cystectomy specimens, was assessed by an experienced pathologist (GC) and is listed together with other clinical data in Additional file 1. The investigation was approved by the local ethical committee at the Lund University and a written consent was obtained from all patients.

### Nucleic acid isolation

DNA was isolated using the DNeasy Tissue Kit (Qiagen, Valencia, CA), including the optional RNase H treatment, and verified for high quality by agarose gel electrophoresis. Total RNA was extracted using Trizol reagent (Invitrogen, Carlsbad, CA). Isolated RNA was purified on Qiagen RNeasy columns (Qiagen) and sample integrities were assessed on an Agilent 2100 Bioanalyzer (Agilent technologies, Palo Alto, CA).

### Microarray hybridizations

Labeling of test and reference DNA was performed as previously described [9], with slight modifications. In brief, 1.5 μg of tumor and male reference DNA was fluorescently labeled with Cy3-dCTP and Cy5-dCTP (Amersham Biosciences, Uppsala, Sweden), respectively, using the Array CGH labeling kit (Invitrogen, Carlsbad, CA), and purified using filter-based spin columns (Cyscribe GFX Purification kit, Amersham Biosciences). Differentially labeled DNA was pooled, mixed with 100 μg Human Cot-1 DNA (Invitrogen), and lyophilized prior to resuspension in 55 μl hybridization solution (50% formamide, 10% dextran sulfate, 2× SSC, 2% SDS, 10 μg/μl yeast tRNA). Probes were heated at 70°C for 15 minutes and at 37°C for 30 minutes before hybridization to micro arrays for 48-72 hours at 37°C. High-resolution tiling BAC arrays produced at the Swegene DNA Micro Array Resource Center, Department of Oncology, Lund University, Sweden [10] using the BAC Re-Array set Ver. 1.0 (32,433 BAC clones) previously described [11] was used. The BAC Re-Array set was obtained from the BACPAC Resource Center at Children's Hospital Oakland Research Institute, Oakland (CA). Prior to hybridization, micro arrays were UV-cross linked at 500 mJ/cm^2 ^and pretreated using the Universal Micro array Hybridization Kit (Corning, Acton, MA) according to the manufacturer's instructions. Slides were washed and scanned as previously described [12]. Oligonucleotide arrays printed with 70-mers from the OPERON v 3.0 set were obtained from the Swegene DNA micro array resource centre [10]. The 36,288 oligonucleotides printed on each slide correspond to 18,466 unique Entrez genes. Sample and Universal Human Reference RNA (Stratagene, La Jolla, CA) labeling, and micro array hybridization was performed using the Pronto Plus System (Promega, Madison, WI; Coring, Acton, MA) according to the manufacturer's specification. Arrays were scanned with an Agilent G2565AA micro array scanner (Agilent technologies).

### Microarray image and data processing

Primary data were collected using the GenePix Pro 4.0 software (Axon Instruments Inc., Foster City, CA), and raw result files were deposited into the web-based database BioArray Software Environment (BASE) [13]. For genomic profiling, spots were background-corrected using the median foreground minus the median background signal intensities for both dyes, and log_2 _ratios were calculated. Unreliable features, marked in the feature extraction software, and spots not showing signal-to-noise ratios ≥ 3, for both channels, were removed. Data normalization was performed per array subgrid using Lowess curve fitting [14] with a smoothing factor of 0.33. Chromosomes X and Y BAC clones were omitted during the estimation of the normalization function. The BASE implementation of CGH-Plotter [15] was used to produce imbalance frequency plots in which BACs were defined as gained, normal, or lost using gain/loss log_2 _ratio thresholds of ± 0.25, and a moving mean sliding window of five clones. For amplification frequency plots, amplifications were defined as regions with at least two consecutive BAC clones showing log_2 _ratios ≥ 1.0. Amplicon sizes were defined as the longest distance between the two outermost amplified BAC clones. In cases of highly discontinuous amplification, amplicons were considered ended when separated by at least two BAC clones with log_2 _ratios below 0.5. Homozygous deletions were defined as regions with consistent log_2 _ratios below -1.2, and with at least one BAC showing a log_2 _ratio < -1.5. Mapping data were obtained from the UCSC genome browser [16]. To identify genomic amplifications specific for G3 tumors a significance analysis of micro arrays (SAM) was performed [17]. Selection of amplification target genes was based on three criteria. Potential target genes should ideally display i) top ranking expression levels in cases with highest DNA copy number of that particular gene, ii) a high Spearman rank correlation throughout the entire data set, and iii) at least 2-fold up regulation when amplified compared to median expression of that gene in the data set.

### Real-time quantitative RT-PCR analysis and genomic PCR analysis

Custom-made TaqMan probes for the test genes and for three internal standards (*ACTB*, *HPRT1*, and *RPLP0*) were obtained from Applied Biosystems (Foster City, CA.), and reactions performed on a Real Time PCR System 7500 (Applied Biosystems) according to the manufacturer's recommendations. To verify potential homozygous deletions sequence-tagged site (STS) markers within the deleted regions were used for semi-quantitative genomic PCR analysis. Quantification of PCR products were performed as previously described [18].

## Results

**Figure 1:**
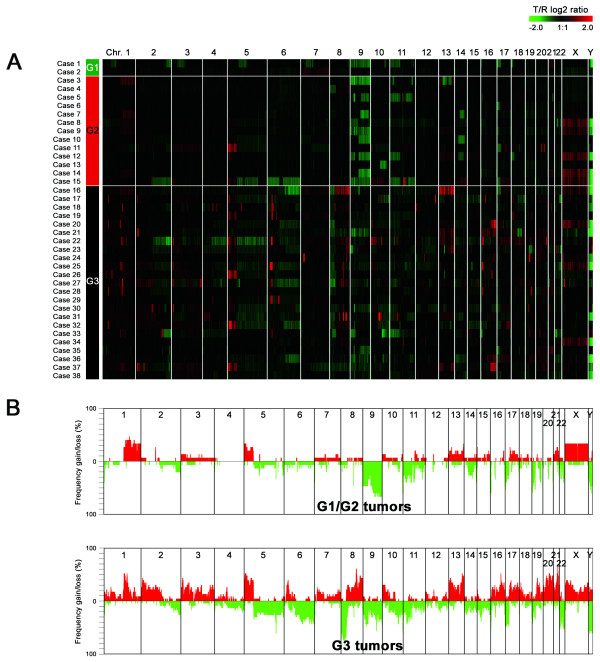
**A**. Genome-wide overview of copy number alterations in 38 bladder tumors. Each row represents a separate tumor sample, with case numbers and tumor grades indicated to the left. Each column shows one of the 32,433 different BAC clones on the microarray, ordered from 1pter to Yqter. Test over reference fluorescence ratios (moving average, symmetric 4-nearest neighbors) based on a log2 pseudocolor scale (indicated) are shown. **B**. Genome-wide imbalance frequency plot for G1/G2 and G3 tumors. Gains and losses are as defined in materials and methods. Red, gains; green, losses.

**Figure 2:**
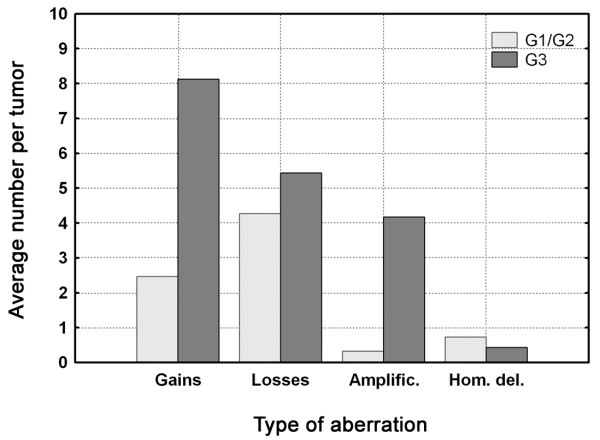
Average number of gains, losses, amplifications and homozygous losses in G1/G2 and G3 tumors, respectively.

Results from the genome-wide DNA copy number analyses of the 38 tumors are shown in Figure 1A (the complete data set is available in Additional file 2). Low and medium grade (G1/G2) tumors generally contained few changes (Figure 1B), paralleled by similar low level of complexity in Ta tumors (Table 1). T1 tumors showed increased frequencies of genomic imbalances, and T2-T4 tumors showed even more complex aberrations with as many as 24 different frequent (> 30%) imbalances. Losses and homozygous deletions were seen at similar frequencies in low/medium and high grade tumors, whereas gains and particularly amplifications were considerably more frequent in G3 tumors (Figure 2); high grade tumors on average showed more than 10 times the number of amplifications as low/medium grade tumors (4.2 vs. 0.3). The most frequent amplifications were localized to 6p22 (8 cases) and 8q22 (5 cases), respectively, both observed exclusively in high-grade tumors. Because only one of the tumors showed co-occurrence of the two events, at least one of these aberrations were present in more than 50% of the G3 tumors (12 of 23 cases).

**Table 1:** Genomic imbalances categorized according to grade and stage^a, b^

**G1/G2**		**G3**		**Ta**		**T1**		**≥ T2**	
**Gains**	**Losses**	**Gains**	**Losses**	**Gains**	**Losses**	**Gains**	**Losses**	**Gains**	**Losses**

+1q	-9	+1q_prox_	5q_prox_-	+1q	9p-	+1q_prox_	6q_dist_-	+1q_prox_	2q_dist_-
+5p_dist_	11p_int_-	+2p_prox_	6q-		9q-	+5p	8p-	+2p_dist_	5q_prox_-
	17p_dist_-	+3p_prox_	8p-			+8q_prox_	9q-	+3p_dist_	6q-
	19p-	+3p_dist_	9p_int_-			+8q_dist_	11p-	+5p	8p-
		+5p	9q_dist_-			+10p	17p-	+6p_int_	9p-
		+6p_int_	11p_int_-			+13	19p-	+8q_int_	9q-
		+8q_int_	17p-			+15q_dist_	22q-	+8q_dist_	11p-
		+8q_dist_	-22			+18p		+10p	17p-
		+10p_dist_				+21q		+13q_prox_	22q-
		+13p_prox_						+13q_dist_	
		+13q_dist_						+16q	
		+16q						+17q	
		+20						+19q_prox_	
		+21q						+20	
								+21q	

^a^Imbalances seen in ≥ 30% of the cases

^b^prox, proximal part of chromosome arm; int, interstitial part of chromosome arm; dist, distal part of chromosome arm.

Apart from the two major targets mentioned above, seven additional segments showed recurrent (≥ 3 cases) overlapping amplifications; 1p34, 1q23, 2p25, 3p25, 10q22, 11q13, and 16p13 (Table 2). The most common of these, 1q23, spanned a 5.4 Mb region with a commonly amplified segment of 0.8 Mb. The genomic profiles of the individual cases were very diverse *e.g*., case 27, with the largest amplification showed the highest amplifications levels in a region 1.2 Mb proximal to the commonly amplified segment, whereas case 28 showed peak log_2 _ratios on the distal side. These findings indicate that 1q23 amplifications are heterogeneous and may represent different genomic events with no common target region. Putative target genes in the other recurrent core amplicons are listed in Table 2. The 11q13 amplification, including *CCND1*, was the only genomic amplification that was recurrent in G1/G2 tumors. A statistical analysis (SAM) confirmed that local amplification of 1q23, 6p22, and 8q22 were significant discriminators between G1/G2 and G3 tumors (data not shown).

**Table 2:** Summary of common recurrently amplified regions^a, b^

**Cytogenetic region**	**Mb start position (BAC)**	**Mb end position (BAC)**	**Size (Mb)**	**Genes**^c^	**No. cases**
1p34	39.27 (RP11-528I12)	40.26 (RP11-747C18)	1.0	*MACF1, PABPC4, TRIT1*	3 (8%)
1q23	157.00 (RP11-735D24)	157.81 (RP11-812N5)	0.8	*-*	4 (11%)
2p25	9.42 (RP11-360P14)	10.34 (RP11-360P14)	0.9		3 (8%)
3p25	10.20 (RP13-635L13)	12.52 (RP11-738A2)	2.3	*TATDN2, SEC13L1, VGLL4*	3 (8%)
6p22	20.07 (RP11-345F7)	22.08 (RP11-630B10)	1.8		8 (21%)
8q22	101.23 (RP11-321E7)	102.99 (RP11-811I18)	1.8		5 (13%)
10q22	76.24 (RP11-368I19)	78.49 (RP11-272P2)	2.3	*MYST4*	3 (8%)
11q13	69.07 (CTD-2192B11)	70.20 (CTD-2011L13)	1.1	*CCND1, ORAOV1, PPFIA1, CTTN*	3 (8%)
16p13	10.59 (RP11-78D17)	12.84 (RP11-310B24)	2.3	*NUBP1, DEXI, TXNDC11, ZC3H7A, RSL1D1, GSPT1*	3 (8%)

^a^Mapping data is based on the UCSC genome browser (May 2004 freeze), and listed regions represent core amplicons.

^b^Only regions amplified in at least 3 cases are listed.

^c^Genes that showed top ranking expression levels when genomically amplified. More detailed data for the 6p22, 8q22, and for 2p25 is given in Additional file 3.

### The 6p22 amplicon

**Figure 3:**
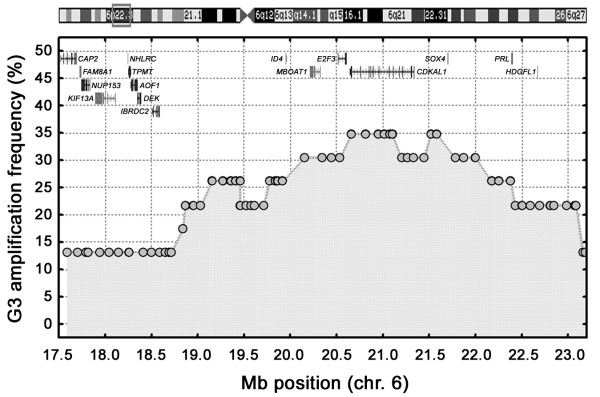
Amplification frequencies in 6p22 among all 23 high-grade tumors. The BAC clones (grey-filled circles) and the genes in the upper part of the graph are positioned/depicted according to their genomic position. The grey square in the ideogram (on top) illustrates the region on chromosome 6 included in the plot.

Genomic amplification in 6p22 was seen in 8 cases. To identify the critical region, BAC clones in the region were classified as amplified or unamplified in all 23 G3 tumors and the amplification frequencies plotted (Figure 3). This delineated a complex 1.8 Mb core amplicon, present in 7-8 (30-35%) of the high grade tumors, and encompassing four genes; *MBOAT1*, *E2F3*, *CDKAL1*, and *SOX4*. Interestingly, only *CDKAL1 *was localized in the region shared by all 8 cases, although some of the proximal exons of the gene extended outside the amplification border in one of the tumors. The core region was flanked by sequences amplified in 5-6 (22-26%) of the high grade tumors; one distal that included *ID4*, and one proximal that included *PRL *and *HDGFL1 *(Figure 3). A 2 Mb segment amplified in three (13%) of the high grade cases was located further distally. Because of the high gene content in this region this latter segment was included in the overall expression analysis of the region. Altogether, expression profiling data were available for 9 of the 16 annotated genes in the commonly amplified segment; *CAP2*, *FAM8A1*, *NUP153*, *KIF13A*, *TPMT*, *AOF1*, *DEK*, *MBOAT1*and *SOX4*.

*NUP153 *and *FAM8A1 *showed a strong association between gene expression and gene copy numbers (Additional file 3). No BAC clones corresponding to the genes *TPMT*, *AOF1 *and *DEK *were available after the final filtration of the BAC array data, however, both *AOF1 *and *DEK *showed substantial correlations between gene expression and gene copy numbers as estimated by BAC clones flanking the genes. The tumors that showed the highest gene copy numbers for *NUP153*, *AOF1 *and *DEK *were also the top ranking cases with regard to expression. In the core amplicon six and four of the seven cases with *SOX4 *and *MBOAT1 *amplifications, respectively, were top ranking with respect to expression. Because no microarray expression data were available for three of the most commonly amplified genes, *E2F3*, *CDKAL1 *and *ID4*, these were analyzed separately by real-time quantitative PCR. This analysis showed a moderate copy number dependent expression for *ID4 *but a strong association between the DNA and mRNA levels for *CDKAL1 *and *E2F3 *(Additional file 4). Hence, a strong link between gene copy numbers and gene expression is seen for the frequently amplified genes *MBOAT1*, *E2F3*, *CDKAL1*, and *SOX4 *as well as for the less frequently amplified *NUP153*, *AOF1, FAM8A1*, and *DEK*.

### The 8q22 and the related 2p25 amplicon

**Figure 4:**
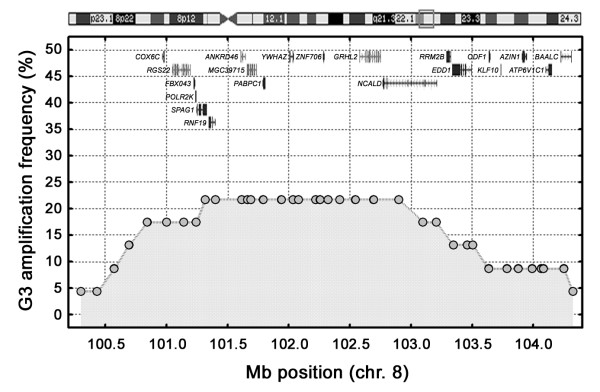
Amplification frequencies in 8q22 among all 23 high-grade tumors. The BAC clones (grey-filled circles) and the genes in the upper part of the graph are positioned/depicted according to their genomic position. The grey square in the ideogram (on top) illustrates the region on chromosome 8 included in the plot.

The core amplicon in 8q22 extended over a 1.8 Mb region and included 10 annotated genes (Figure 4), of which expression data were available for five, *POLR2K, RNF19, PABPC1, YWHAZ*, and *NCALD *(Additional file 3). For two of these, *POLR2K *and *YWHAZ *(*14-3-3*-*zeta*), the tumors with highest copy numbers also showed the highest expression. Furthermore, these genes showed Spearman rank correlations of 0.77 and 0.68, respectively, between gene expression and gene copy numbers suggesting a strong link between *POLR2K *and *YWHAZ *gene copy numbers and expression. Three additional tumor cases, all G3 tumors, shared a 1 Mb amplified region in 2p25 (Additional file 3). A comprehensive analysis revealed that this segment contained several genes paralogous to genes in the 8q22 amplicon; *YWHAZ, GRHL2*, *NCALD*, *RRM2B*, and *KLF10 *in 8q22 showed the related genes *YWHAQ *(*14-3-3-theta*), *GRHL1*, *HPCAL1*, *RRM2*, and *KLF11 *in the 2p25 amplicon. Among the 13 genes in the 2p25 amplicon for which expression data were available, six genes showed top ranking expression levels when amplified, *ITGB1BP1*, *CPSF3*, *ADAM17*, *YWHAQ*, *TAF1B *and *RRM2*. Hence, the common denominator between the 8q22 and the 2p25 amplicons is the increase in expression of the *14-3-3-*genes. Intriguingly, none of the cases with 8q22 amplifications showed concurrent amplification of 2p25. The seemingly complementing 8q22 and 2p25 amplifications were thus seen in the same frequency as the 6p22 amplifications.

### Homozygous deletions

**Figure 5:**
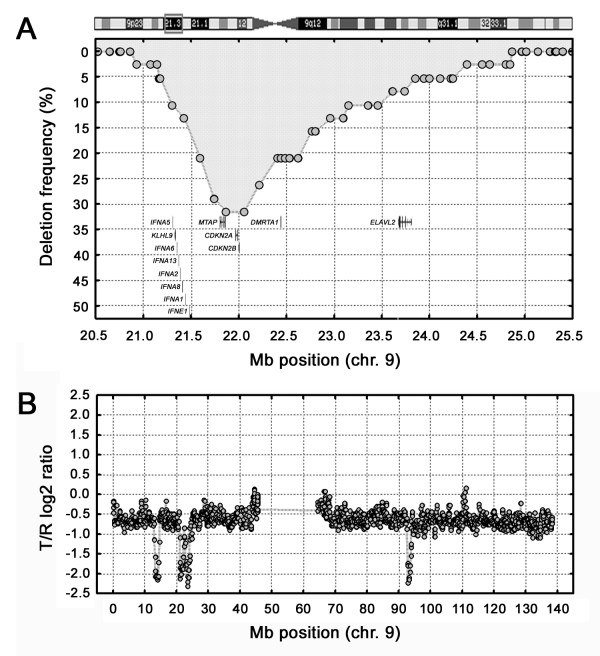
**A**. Homozygous deletion frequencies in 9p21 among all 38 tumors. The BAC clones (grey-filled circles) and the genes in the lower part of the figure are positioned/depicted according to their genomic position. **B**. Gene copy number profile of chromosome 9 in case 3. The profile shows three separate homozygous deletions in the otherwise heterozygously deleted chromosome.

The array CGH analysis indicated homozygous deletion in ten different genomic locations of which six were verified by semi-quantitative Q-PCR (Additional file 5). The most frequent was observed in 9p21, detected in 12 (32%) of the cases (Figure 5A). These deletions included the *CDKN2A *(*p14*^*ARF*^), *CDKN2B*, and *MTAP *loci in all cases and spanned a 4 Mb region, mainly extending at the proximal side of the *CDKN2A/CDKN2B *loci. The frequency of 9p21 homozygous deletion did not differ between G1/G2 and G3 cases (χ^2^, p > 0.05). Interestingly, homozygous loss of the *CDK2A/CDK2B *locus was not seen in cases with 6p22 amplifications, indicating that these changes are not independent events (p = 0.017, Fishers exact test). Homozygous deletions were detected in five additional locations on chromosome 9; in 9p24, 9p23, 9q21, 9q22, and in 9q33 respectively, of which the 9q22 deletion was detected in two cases. Case 3 was exceptional as this tumor showed three different homozygous deletions on chromosome 9, in 9p23, 9p21 and in 9q21 (Figure 5B). Additional homozygous deletions were detected in 4q35, 10q26, 13q14, and 21q21, of which only one covered a known tumor suppressor gene, *RB1 *in 13q14.

## Discussion

We performed whole-genome tiling-resolution array CGH analyses to characterize gene copy number aberrations in 38 cases of urothelial carcinomas. To make a comprehensive evaluation of the consequences at the expression level the array CGH data was compared with expression data obtained by high density expression profiling. Low grade and early stage tumors showed a limited number of genomic imbalances with gain of 1q and loss of chromosome 9q as the most frequent changes. T1 tumors harbored additional changes and frequently showed losses of 6q, 8p, 9q, 11p, 17p, 19p, and 22q, and gains of 1q, 5p, 8q, 10p, and 13, and 5p. Late-stage tumors, T2-T4, showed several additional genomic imbalances and showed complex genomic changes. A major difference was seen between G1/G2 and G3 tumors with regard to gains and amplifications. Gains were more frequent in G3 tumors and amplifications more than 10 times as frequent in high grade than in low/medium grade tumors. This is in line with the recent finding that high grade tumors show frequent anaphase bridges that ultimately may lead to breakage-fusion-bridge cycles producing genomic amplifications [19]. In contrast to Blaveri et al. [7], we did not see a reduction in the number of affected regions in late-stage tumors. Instead T2-T4 tumors showed considerably more complex changes than Ta/T1 tumors, corresponding to previous cytogenetic and conventional CGH results that have shown a larger number of genomic alterations in high-grade and late-stage tumors [2-4,20].

The analysis showed that amplifications of 1q23 were highly heterogeneous. Individual cases showed extended amplicons with peak copy number ratios in different areas and with no obvious commonly amplified segment. Gain of chromosome arm 1q is frequent in UC [1,2] and local amplifications in 1q23 have previously been reported in studies using conventional CGH [3,5]. However, due to limited resolution in traditional CGH analysis diverse amplifications within a 5 Mb genomic region may have been interpreted as a common genomic event and hence assumed to target the same genomic segment. Interestingly, the present tiling resolution analysis indicates that this is most likely not the case. There are at least two possible explanations for these findings; either the 1q23 region harbors more than one target segment, or the 1q23 region show local genomic instability resulting in frequent gains or high level amplifications. Both situations would result in a heterogeneous pattern of changes.

The most frequently amplified chromosomal segment was 6p22. This segment has been repeatedly shown to be amplified in UC and to cover the genes *E2F3*, *CDKAL1*, and *SOX4 *[6,8,21-25]. In the present investigation a 1.8 Mb core region, included in 88-100% of the cases with 6p22 amplifications, was defined. This region contains *MBOAT1, E2F3*, *CDKAL1*, and *SOX4*. The core segment was flanked by two regions included in 38-75% of the amplifications that on the distal side contained *ID4*, and on the proximal side *PRL *and *HDGFL1*. A further distally located region, present in three (38%) of the cases with amplification, contained ten additional genes. In total a more than 6 Mb region was found to be repeatedly included in the 6p22 amplifications. To identify possible targets genes within this segment we used different criteria based on available expression data. Six genes; *NUP153*, *AOF1*, *DEK, SOX4, FAM8A1*, and *MBOAT1 *showed top ranking expression levels when amplified. By Q-PCR we also established a strong association between *E2F3 *and *CDKAL1 *gene expression and gene copy numbers. This identifies eight possible candidate genes in 6p22 that may be ranked according the frequency by which they take part in 6p22 amplifications as *CDKAL1*, *E2F3*, and *SOX4 *> *MBOAT1 *> *DEK*, *NUP153, FAM8A1*, and *AOF1*.

Several investigations have shown a strong association between gene copy numbers and gene expression for *E2F3 *and *CDKAL1 *[8,21,22]. In two recent studies knock down experiments of *E2F3 *in bladder cancer cells lines with 6p amplification was shown to strongly reduce cellular proliferation whereas a similar knock down of *CDKAL1 *did not have this effect [26,27]. This indicates *E2F3 *as a major target for the 6p22 amplification. The *E2F3 *locus produces two alternative proteins, E2F3a and E2F3b [28]. E2F3a is an inducible activator of cell cycle progression whereas E2F3b is constitutively expressed and functions as a negative regulator of *p14*^*ARF *^expression, thereby compromising TP53 activity [29]. Genomic amplification of the *E2F3 *locus may consequently contribute to tumor development both by accelerating the cell cycle and by raising the threshold for apoptosis via inactivation of *p14*^*ARF*^. In this context it is worth noting that none of the cases with 6p amplification showed homozygous deletions leading to loss of *p14*^*ARF*^. Hence, amplification of 6p and homozygous deletion of the *CDKN2A/CDKN2B *locus behaves as complementary events; one leading to the simultaneous loss of *CDKN2A*, a cell cycle inhibitor, and *p14ARF *activity, and a second to increased *E2F3*, a cell cycle activator, and reduced *p14*^*ARF *^activity. As a consequence 65% of the G3 tumors may show impaired *p14*^*ARF *^function.

*SOX4 *belongs to the SRY-related HMG-box (SOX) family of transcription factors involved in the regulation of embryonic development and in cell fate determination. The protein acts as a transcriptional regulator in the apoptosis pathway as well as in pathways leading to tumorigenesis. *SOX4 *is over expressed in several tumor types [30] and was found to be significantly over expressed in bladder tumors in a recent investigation [31], suggesting a role for *SOX4 *in bladder cancer development. Intriguingly, both experimentally increased [31] and decreased [32]*SOX4 *expression induces an apoptotic response. This may indicate that *SOX4 *expression has to be finely tuned not to induce deleterious cellular responses and may, partly, explain why no consistent correlation between *SOX4 *amplification and expression has been seen in previous investigations [8,21,31].

The *MBOAT1, DEK*, *FAM8A, AOF1*, and *NUP153 *genes were amplified at lower frequencies than *E2F3*. Of these *DEK*, a chromatin-associated protein could provide a selective advantage when amplified as DEK function as an inhibitor of senescence [33]. Furthermore, as *DEK *is under the transcriptional control of E2F3 [34] co-amplification of *DEK *with *E2F3 *may enhance *DEK *expression further. NUP153 plays an important role in nuclear pore function and have key functions in both import and export to the nucleus. NUP153 is known to affect the sub cellular distribution of transcription factor such as SMAD2, STAT1, and PU.1 [35], hence, altered *NUP153 *expression may modify the accessibility of key regulatory proteins. The possible roles of *MBOAT1*, *FAM8A1*, and *AOF1 *in carcinogenesis are, however, less clear. Taken together, the present comprehensive analysis of 6p22 amplification in UC indicates that *SOX4*, *DEK *and *NUP153 *may contribute to cellular transformation when co-amplified with *E2F3*, and hence that 6p22 may represent a composite amplicon with more than one potential target gene.

The core amplicon in 8q22 extended over a 1.8 Mb region and included 8 annotated genes of which *YWHAZ *(*14-3-3*-*zeta*) and *POLR2K *showed a strong association between gene copy number and gene expression. Interestingly, three tumors with no amplification of 8q22 showed amplification of a 1 Mb paralogous region in 2p25. This segment included several genes similar to genes within the 8q22 segment, such as *YWHAQ*, *GRLH1*,*HCAL1*, *RRM2*, and *KLF11*. Two of the paralogous genes within the 2p25 amplicon showed a strong association between gene copy numbers and gene expression, *YWHAQ *(*14-3-3-theta*) and *RRM2*. Hence, the common denominator of the 8q22 and 2p25 amplicons is the amplification and over expression of the paralogous *14-3-3*-*zeta *and *14-3-3*-*theta *genes. Furthermore, if amplification of the *YWHA *genes may substitute for each other, amplification of these genes is as common as amplification of *E2F3*.

There are seven 14-3-3 isoforms described in mammals [36]. The proteins are widely expressed and bind as dimers to client proteins thereby modulating their enzymatic activity, sub cellular localization, or potential to form protein complexes; more than 200 proteins have been reported to associate with these proteins. It is believed that 14-3-3 proteins functions as general survival factors by enhancing pro-survival signaling and suppressing pro-apoptotic proteins [36]. The findings that *14-3-3*-*zeta *and -*theta *show increased expression in oral squamous cell carcinomas and lung, stomach, and breast cancers [37-40], and that mice transgenic for *14-3-3*-*zeta *develop various types of tumours at young age [41] favors the conclusion that *14-3-3*-*zeta *and -*theta *may act as oncogenes also in urothelial carcinomas.

Homozygous deletions were detected in six locations on chromosome 9 and in four locations in the remaining genome; 4q35, 10q23, 13q14, and 21q21. None of the observed deletions coincides with deletions frequently seen in cell lines [42] and only one, 9p21, has previously been reported for urothelial carcinomas. Among the deletions not occurring on chromosome 9 three, 4q35, 10q23, and 21q21 respectively, extended over genes not previously associated with tumor development and may merit further analyses. The 13q14 deletion, on the other hand, affected the known tumor suppressor gene *RB1*, and hence most likely has a pathological effect. As expected the most frequent homozygous deletions occurred at 9p21, in a 4 Mb segment that consistently included *CDKN2A *and *CDKN2B*. The observed frequency of *CDKN2A *homozygous deletions reached 32%, which is close to the frequency found by Q-PCR of micro dissected tumor biopsies [43]. The deletions were more extensive on the proximal than on the distal side of *CDKN2A *which may be caused by the fact that the proximal region is less dense with genes. An intriguing finding was the clustering of homozygous deletions to chromosome 9. Homozygous deletions were seen in five chromosome 9 locations, excluding the high-frequency *CDKN2A *region, compared with a total of four homozygous deletions on other chromosomes. Furthermore, one case showed two homozygous deletions, and another case one homozygous deletion in addition to a 9p21 deletion. This high frequency of homozygous deletions is astonishing but is in agreement with the high frequency of LOH and chromosomal 9 losses seen in UC [44]. The accumulated data reveals no specific pattern of LOH, which has led to the suggestion that most of the LOH seen in chromosome 9 may be caused by unspecific mitotic recombination [45]. A possible mechanism for the frequent homozygous deletions could consequently be unequal mitotic recombination events. Such events would produce homozygous deletions after segregation of the recombined homologues, as well as segmental duplications. Indeed, possible segmental duplications on chromosome 9 were seen in a limited number of cases. Irrespective of the mechanisms causing the deletions, the high frequency of homozygous deletions at various locations on chromosome 9 point to the possibility that these deletions may have a different origin and cellular consequence than the more frequent 9p21 homozygous deletions.

## Conclusion

The presented data indicates 6p22 as a composite amplicon with more than one possible target gene and suggests that amplification of 6p22 and homozygous deletions of 9p21 may have complementary roles. The combined data for amplified 8q22 and 2p25 paralogous genomic segments indicated that alterations of *YWHA *(14-3-3) genes may be important in the development of urothelial carcinomas. Furthermore, chromosome 9 show an exceptionally high frequency of homozygous deletions compared to other chromosomes.

## Competing interests

The author(s) declare that they have no competing interests.

## Authors' contributions

MHe performed the array-CGH analyses and took active part in the bioinformatical and statistical analyses, and in preparing the manuscript; DL performed the expression profiling and took active part in the bioinformatical and statistical analyses as well as preparing the manuscript, TJ performed the Q-PCR analyses; SV performed bioinformatic analyses; FL, SG, and WM provided tumor samples and clinical data; GC performed the pathological evaluations; ÅB provided the array-CGH platform and technical assistance; MHo conceived the investigation and took active part in the bioinformatical and statistical analyses as well as preparing the manuscript. All authors read and approved the manuscript.

## Pre-publication history

The pre-publication history for this paper can be accessed here:



## Supplementary Material

Additional file 1 Clinical data. Data on sex, age, stage, and grade given for each patient as well as which samples were analyzed by expression profiling. Click here for file

Additional file 2 Array-CGH data. BAC clone, genes, cytoband, start and end of BAC clone are given as well as the gene copy ratios in the individual samples. Click here for file

Additional file 3 Correlations between gene copy numbers and gene expression. The correlation between gene copy numbers and gene expression for genes within the 2p25, 6p22, and 8q22 amplicons. Click here for file

Additional file 4 *ID4*, *E2F3*, and *CDKLA1 *gene expression. Results from Q-PCR analyses of *ID4*, *E2F3*, and *CDKLA1 *gene expression. Click here for file

Additional file 5 Homozygous deletions. Table of cytogenetic and Mbp positions, sizes, and included genes in the identified homozygous deletions. Click here for file
