# Supplementary figures and images for: Tiling resolution array CGH and high density expression profiling of urothelial carcinomas delineate genomic amplicons and candidate target genes specific for advanced tumors
Source: BMC Med Genomics. 2008 Jan 31;1:3. doi: 10.1186/1755-8794-1-3 (PMC2227947; doi:10.1186/1755-8794-1-3)

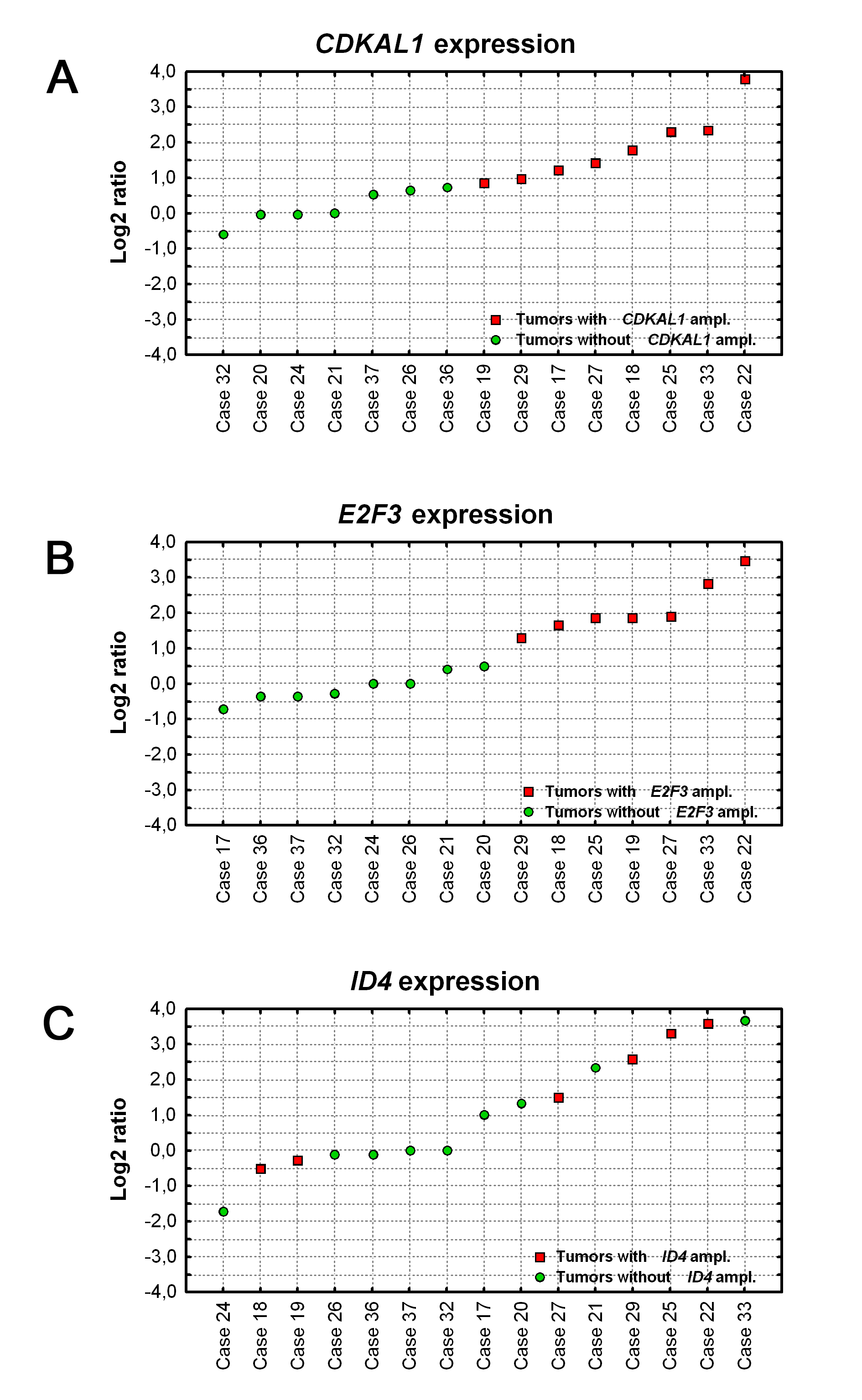

Supplement: Additional file 1 — Clinical data. Data on sex, age, stage, and grade given for each patient as well as which samples were analyzed by expression profiling. [file 1755-8794-1-3-S1.TIFF]
