# Supplementary material for: Tiling resolution array CGH and high density expression profiling of urothelial carcinomas delineate genomic amplicons and candidate target genes specific for advanced tumors
Source: BMC Med Genomics. 2008 Jan 31;1:3. doi: 10.1186/1755-8794-1-3 (PMC2227947; doi:10.1186/1755-8794-1-3)
Supplement: Additional file 2 — Array-CGH data. BAC clone, genes, cytoband, start and end of BAC clone are given as well as the gene copy ratios in the individual samples. [file 1755-8794-1-3-S2.DOC]

**Supplementary Table 1.**

Clinical data on the 38 patients with urothelial carcinomas

| **Case no.**  **(Lab no.)** | **Sex/Age** | **Stage/Grade** | **35k oligo**  **data** |
| --- | --- | --- | --- |
| 1. (3853-01) | M/64 | Ta/G1 | - |
| 2. (3599-02) | M/45 | Ta/G1 | - |
| 3. (905-01) | M/55 | Ta/G2 | Yes |
| 4. (1411-01) | M/88 | Ta/G2 | - |
| 5. (1893-01) | M/75 | Ta/G2 | Yes |
| 6. (3585-01) | M/73 | Ta/G2 | - |
| 7. (1785-02) | F/38 | Ta/G2 | - |
| 8. (1857-02) | F/81 | Ta/G2 | - |
| 9. (3132-02) | M/70 | Ta/G2 | - |
| 10. (478-02) | M/72 | Ta/G2 | Yes |
| 11. (904-01) | M/71 | T1/G2 | - |
| 12. (1780-02) | F/81 | T1/G2 | Yes |
| 13. (2268-02) | M/81 | T1/G2 | - |
| 14. (3517-02) | F/82 | T1/G2 | Yes |
| 15. (2646-01) | F/74 | T1/G3 | Yes |
| 16. (16-02) | M/58 | T1/G3 | Yes |
| 17. (1619-03) | M/52 | T1/G3 | Yes |
| 18. (1269-04) | M/75 | T1/G3 | Yes |
| 19. (4652-04) | F/65 | T1/G3 | Yes |
| 20. (1261-04) | F/50 | T2/G2 | Yes |
| 21. (247-01) | M/57 | T2/G3 | Yes |
| 22. (684-01) | M/59 | T2/G3 | Yes |
| 23. (1811-01) | M/68 | T2/G3 | Yes |
| 24. (2360-01) | F/74 | T2/G3 | Yes |
| 25. (3583-01) | F/64 | T2/G3 | Yes |
| 26. (315-02) | M/63 | T2/G3 | Yes |
| 27. (876-02) | M/69 | T2/G3 | Yes |
| 28. (879-02) | M/63 | T2/G3 | Yes |
| 29. (1681-02) | M/71 | T2/G3 | Yes |
| 30. (2766-02) | M/66 | T2/G3 | Yes |
| 31. (3755-02) | M/71 | T2/G3 | Yes |
| 32. (1301-03) | M/76 | T2/G3 | Yes |
| 33. (1695-03) | M/60 | T2/G3 | Yes |
| 34. (2402-03) | F/69 | T2/G3 | Yes |
| 35. (2403-03) | M/46 | T2/G3 | Yes |
| 36. (1805-04) | F/68 | T2/G3 | Yes |
| 37. (1909-04) | M/66 | T2/G3 | Yes |
| 38. (2130-04) | M/83 | T2/G3 | Yes |
