# Supplementary material for: Tiling resolution array CGH and high density expression profiling of urothelial carcinomas delineate genomic amplicons and candidate target genes specific for advanced tumors
Source: BMC Med Genomics. 2008 Jan 31;1:3. doi: 10.1186/1755-8794-1-3 (PMC2227947; doi:10.1186/1755-8794-1-3)
Supplement: Additional file 5 — Homozygous deletions. Table of cytogenetic and Mbp positions, sizes, and included genes in the identified homozygous deletions. [file 1755-8794-1-3-S5.doc]

**Supplementary table 3.** Summary of homozygous deletions^a,b^

| **Cytogenetic region** | **Mb start position (BAC)** | **Mb end position (BAC)** | **STS**^b^ | **Size (Mb)** | **Genes** | **No. cases** |
| --- | --- | --- | --- | --- | --- | --- |
| 4q35 | 189.46 (RP11-156B8) | 189.76 (CTD-2314A8) | SHGC-140848 | 0.30 | *FLJ25801, FLJ36180* | 1 |
| 9p24 | 0.24 (RP11-393D13) | 0.86 (RP11-696A8) |  | 0.62 | *DOCK8, LOC645586, LOC642350, ANKRD15, DMRT1* | 1 |
| 9p23 | 13.12 (RP11-173M6) | 14.53 (RP11-713D18) | SHGC-140269 | 1.41 | *MPDZ, NFIB, LOC646181, FLJ41200,*  *LOC646206, LOC646211, LOC347193, LOC138864,* | 1 |
| 9p21^c^ | 21.75 (RP11-615P15) | 22.15 (RP11-467K20) | SHGC-9726 | 0.40 | *MTAP, CDKN2A, CDKN2B* | 12 |
| 9q21 | 74.84 (RP11-714O6) | 75.18 (RP11-715L22) | SHGC-82463 | 0.34 | *OSTF1, NRK1, C9orf41, C9orf40* | 2 |
| 9q22 | 92.76 (RP11-612N7) | 93.87 (RP11-275N7) | SHGC-130828 | 1.11 | *FGD3, SUSD3, BINCA, NINJ1, WNK2,*  *C9orf10OS, C9orf10, PHF2, BARX1* | 1 |
| 9q33^b^ | 122.86 (RP11-102I1) | 124.10 (RP11-160B11) | SHGC-130269 | 1.24 | *RABGAP1, GPR21, STRBP, CRB2, DENND1A, LHX2, NEK6, PSMB7* | 1 |
| 10q26^b^ | 134.20 (RP13-502M1) | 134.53 (RP11-33G5) |  | 0.33 | *NKX6-2* | 1 |
| 13q14 | 47.90 (RP11-115I22) | 50.40 (RP11-686G10) |  | 2.50 | *RB1, RCBTB2, CYSLTR2, FNDC3A, CDADC1, MLNR, CAB39L, RCBTB1, SETDB2, PHF11, CAB39L, RCBTB1, ARL11, EBPL, KPNA3, C13orf1, KCNRG, DLEU2, RFP2, DELEU1, DLEU2,FAM10A4, DLEU7, FLJ11712* | 1 |
| 21q21 | 16.03 (RP11-268F23) | 17.63 (RP11-486H22) |  | 1.60 | *USP25, VDAC2P, C21orf34* | 1 |

^a^Mapping data is based on the UCSC genome browser (May 2004 freeze)

^b^STS markers used for verification.

^c^Regions where not all cases passed the defined threshold for homozygous deletions, but where a manual data review strongly suggested loss of both alleles.
